# Tip-DC Development during Parasitic Infection Is Regulated by IL-10 and Requires CCL2/CCR2, IFN-γ and MyD88 Signaling

**DOI:** 10.1371/journal.ppat.1001045

**Published:** 2010-08-12

**Authors:** Tom Bosschaerts, Martin Guilliams, Benoît Stijlemans, Yannick Morias, Daniel Engel, Frank Tacke, Michel Hérin, Patrick De Baetselier, Alain Beschin

**Affiliations:** 1 Department of Molecular and Cellular Interactions, VIB, Brussels, Belgium; 2 Laboratory of Cellular and Molecular Immunology, Vrije Universiteit Brussel, Brussels, Belgium; 3 Institute for Molecular Medicine and Experimental Immunology, University Clinic of Bonn, Bonn, Germany; 4 Department of Medicine III, RWTH-University Hospital Aachen, Aachen, Germany; 5 Cell and Tissue Laboratory, Unité de Recherche en Physiologie Moléculaire, Facultés Universitaires Notre-Dame de la Paix, Namur, Belgium; University of Wisconsin-Madison, United States of America

## Abstract

The development of classically activated monocytic cells (M1) is a prerequisite for effective elimination of parasites, including African trypanosomes. However, persistent activation of M1 that produce pathogenic molecules such as TNF and NO contributes to the development of trypanosome infection-associated tissue injury including liver cell necrosis in experimental mouse models. Aiming to identify mechanisms involved in regulation of M1 activity, we have recently documented that during *Trypanosoma brucei* infection, CD11b^+^Ly6C^+^CD11c^+^ TNF and iNOS producing DCs (Tip-DCs) represent the major pathogenic M1 liver subpopulation. By using gene expression analyses, KO mice and cytokine neutralizing antibodies, we show here that the conversion of CD11b^+^Ly6C^+^ monocytic cells to pathogenic Tip-DCs in the liver of *T. brucei* infected mice consists of a three-step process including (i) a CCR2-dependent but CCR5- and Mif-independent step crucial for emigration of CD11b^+^Ly6C^+^ monocytic cells from the bone marrow but dispensable for their blood to liver migration; (ii) a differentiation step of liver CD11b^+^Ly6C^+^ monocytic cells to immature inflammatory DCs (CD11c^+^ but CD80/CD86/MHC-II^low^) which is IFN-γ and MyD88 signaling independent; and (iii) a maturation step of inflammatory DCs to functional (CD80/CD86/MHC-II^high^) TNF and NO producing Tip-DCs which is IFN-γ and MyD88 signaling dependent. Moreover, IL-10 could limit CCR2-mediated egression of CD11b^+^Ly6C^+^ monocytic cells from the bone marrow by limiting *Ccl2* expression by liver monocytic cells, as well as their differentiation and maturation to Tip-DCs in the liver, showing that IL-10 works at multiple levels to dampen Tip-DC mediated pathogenicity during *T. brucei* infection. A wide spectrum of liver diseases associates with alteration of monocyte recruitment, phenotype or function, which could be modulated by IL-10. Therefore, investigating the contribution of recruited monocytes to African trypanosome induced liver injury could potentially identify new targets to treat hepatic inflammation in general, and during parasite infection in particular.

## Author Summary

Most infections are associated with host inflammatory responses that can result in multiple organ failure and death. It is therefore essential to understand the mechanisms balancing host immune response and tissue damage. Mouse models of African trypanosome infection represent valuable tools to study the mechanisms contributing to the inflammatory (pathogenic) or anti-inflammatory (anti-pathogenic) immune response. We recently identified TNF and NO producing DCs (Tip-DCs) as major contributors to liver pathogenicity in *Trypanosoma brucei* infected mice. Herein, the role of different chemokine and cytokines in the generation of Tip-DCs was investigated. Tip-DCs originated from bone marrow derived monocytes that egressed to the blood in a CCR2 chemokine receptor dependent manner. Then, monocytes extravasated to inflamed liver where IFN-γ and MyD88 signaling promoted their maturation to Tip-DCs. Both the egression of monocytes from bone marrow and their IFN-γ/MyD88 dependent maturation to Tip-DCs was counteracted by IL-10, hereby reducing liver pathogenicity. Liver injury, affecting millions of persons worldwide with often lethal consequences, frequently results from uncontrolled activation of recruited monocyte-derived cells that can be modulated by IL-10. Thus, the mechanisms regulating liver immunopathogenicity during parasitic infection identified herein could lead to new therapeutic policies in the field of hepatic inflammation.

## Introduction

Inflammatory immune responses against invading pathogens require the recruitment of immune cells to the site of infection. However, the infiltration of infected tissue by activated immune cells may result in tissue injury justifying a profound understanding of the mechanisms underlying the recruitment and activation of inflammatory cells. Experimental infections with African trypanosomes, extracellular blood-borne parasites that cause sleeping sickness in humans and Nagana disease in cattle in sub-Saharan Africa [1], [2], are used as model systems to study infection-associated liver pathogenicity [3]. In murine models, the control of parasitemia is mostly mediated in the liver by IFN-γ- and MyD88-dependent generation of classically activated monocytic cells (M1) that secrete trypanotoxic molecules TNF and NO and exert phagocytic activity [4], [5], [6], [7]. Within M1, CD11b^+^Ly6C^+^CD11c^+^ inflammatory DCs have been identified as the main population producing TNF and NO during *Trypanosoma brucei* infection [8]. These TNF and iNOS producing DCs (Tip-DCs) originated from bone marrow CD11b^+^Ly6C^+^ monocytic cells recruited to the liver, spleen and lymph nodes of infected mice. On the other hand, the production of TNF and NO contributes to infection-associated pathogenicity including liver cell apoptosis/necrosis, resulting in organ failure and thus negatively affecting survival of the *T. brucei* infected host [3]. In this respect, IL-10 was shown to limit liver inflammation/injury and prolong survival by dampening the IFN-γ producing activity of T cells [9], [10] and by directly limiting the differentiation of CD11b^+^Ly6C^+^ monocytic cells to functional Tip-DCs [8]. Furthermore, IL-10 triggers the expression of genes associated with alternative activation of monocytic cells (M2) that could contribute to anti-inflammatory and wound-healing processes during African trypanosome infection [10], [11].

Since the recruitment of bone marrow derived CD11b^+^Ly6C^+^ monocytic cells to inflamed tissue and their subsequent differentiation in M1-type, TNF and NO producing CD11b^+^Ly6C^+^CD11c^+^ Tip-DCs may have a negative impact on the outcome of *T. brucei* infection, interfering with their recruitment to inflamed tissue could lead to new anti-disease treatments. Therefore we scrutinized the potential pathways governing the recruitment of inflammatory CD11b^+^Ly6C^+^ monocytic cells to the liver as well as their subsequent differentiation to functional Tip-DCs during *T. brucei* infection.

## Results

### Induction of chemokines in the liver of *T. brucei* infected mice

**Figure 1:**
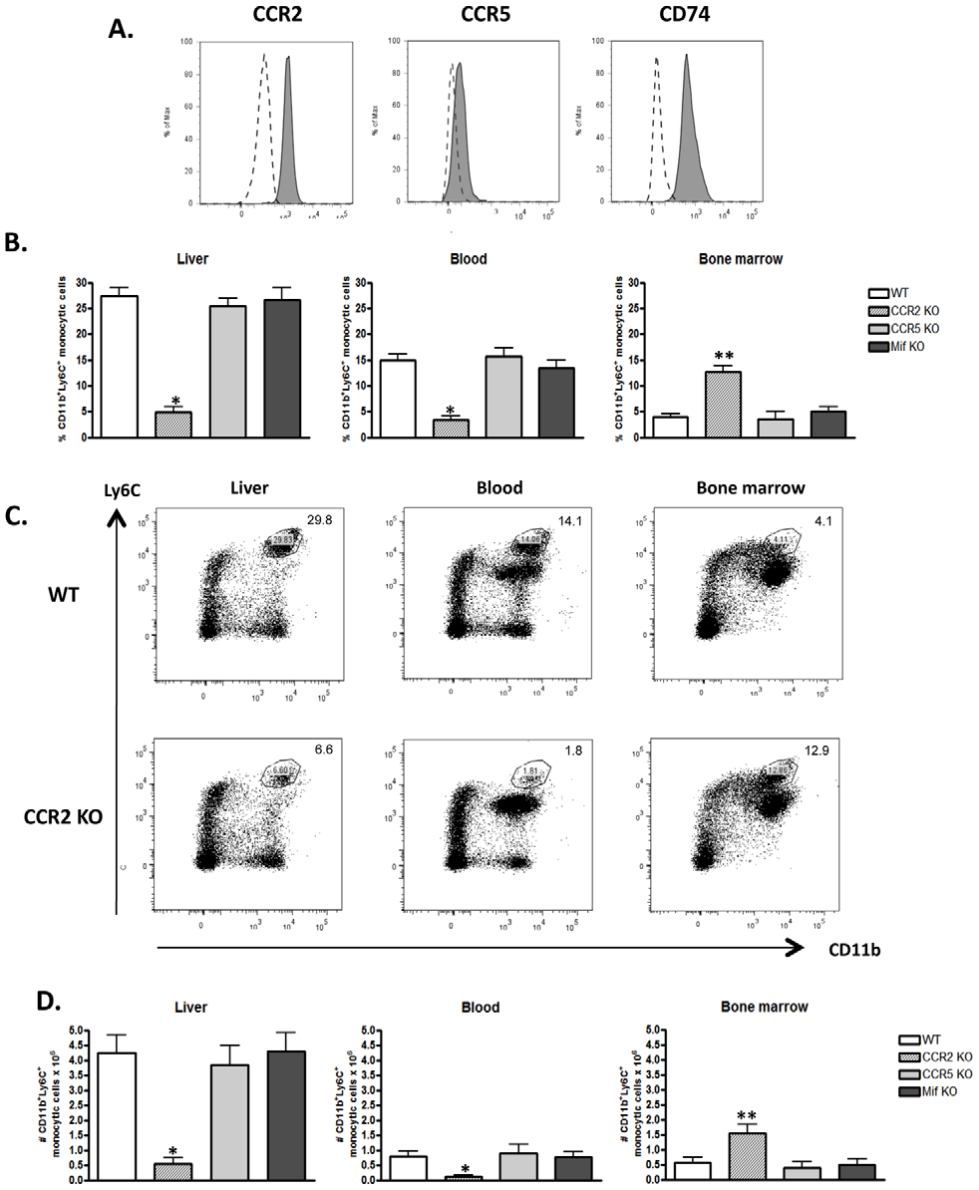
CD11b^**+**^Ly6C^**+**^ monocytic cells are affected by CCR2 but not CCR5 or Mif signaling during *T. brucei* infection. A) Inflammatory monocytic cells gated based on their expression of CD11b and Ly6C from non-parenchymal cells isolated from the liver of WT mice on day 6 of *T. brucei* infection were stained for CCR2, CCR5 or CD74 expression (filled grey curves compared to dotted line curves representing specific isotype controls) FACS profiles are representative of one of four animals tested in two independent experiments. B) Percentage and D) Number of CD11b^+^Ly6C^+^ monocytic cells in liver non-parenchymal, blood and bone marrow of CCR2 KO, CCR5 KO and Mif KO mice on day 6 post infection. Data are shown as mean ± SEM of three individual mice of one of three independent experiments performed. C) Non-parenchymal cells isolated from liver, blood and bone marrow of WT and CCR2 KO mice on day 6 post infection were assayed for co-expression of CD11b and Ly6C. Percentages of CD11b^+^Ly6C^+^ monocytic cells within the indicated gate are shown. FACS profiles are representative of one of nine animals tested in three independent experiments. *, significantly lower (*p*<0.01) and **, significantly higher (p<0.05) compared to WT mice.

*T. brucei* infection in C57Bl/6 mice is characterized by a massive expansion of CD11b^+^Ly6C^+^ monocytic cells in the liver [8]. These cells are closely related to CD11b^+^Ly6C^+^CCR2^+^ inflammatory monocytes that are recruited in infected tissues during microbial infection [12]. To identify pathway(s) possibly involved in the recruitment of liver CD11b^+^Ly6C^+^ monocytic cells, a custom in-house developed mRNA array was used to screen for the expression of chemokine genes in total liver extracts at day 6 post infection, i.e. when the first most important peak of parasitemia and the highest expansion of CD11b^+^Ly6C^+^ monocytic cells in the liver (reaching 27.8±2.5% within liver non-parenchymal cells in infected animals versus 2.1±0.3% in non-infected mice) occur [8]. Seven chemokine genes were found to be significantly induced (more than 2-fold as compared to gene expression in total liver extracts from non-infected mice) and their expression in total liver extracts was subsequently confirmed by RT-PCR (Table 1). The identified genes included (i) T cell-attracting chemokine genes; *Cxcl10* (IP-10) and *Cxcl9* (Mig) acting through the CXCR3 receptor and (ii) monocyte-attracting chemokine genes; *Ccl3* (MIP-1α), *Ccl4* (MIP-1β) and *Ccl5* (RANTES), all ligands for the CCR5 chemokine receptor; *Ccl2* (MCP-1), the preferential ligand for chemokine receptor CCR2; and *Mif* that interacts predominantly with the CD74 receptor but also potentially with CXCR2 and CXCR4. These data raised the possibility that signaling through CCR5 or CCR2 chemokine receptor or through Mif may be involved in the recruitment of liver associated CD11b^+^Ly6C^+^ monocytic cells during *T. brucei* infection. This hypothesis was supported by the observation that liver CD11b^+^Ly6C^+^ monocytic cells expressed CCR2, CCR5 and CD74 on their surface, although at different levels (Fig. 1A).

**Table 1:** Chemokine mRNA expression levels in total liver extracts from *T. brucei* infected mice as determined by real-time PCR.

Chemokine	Fold induction a	Receptor(s)
*Cxcl9* (MIG)	4.4±0.8	CXCR3
*Cxcl10* (IP-10)	9.7±1.2	CXCR3
*Ccl3* (MIP-1α)	4.5±0.6	CCR5, CCR1
*Ccl4* (MIP-1β)	10.5±1.7	CCR5
*Ccl5* (RANTES)	5.6±0.8	CCR5, CCR1, CCR3
*Ccl2* (MCP-1)	5.3±0.9	CCR2
*Mif*	11.2±1.3	CD74, CXCR2, CXCR4

a on day 6 post infection, normalized with the ribosomal protein S12 gene and expressed relative to liver extract from non-infected mice. Data are shown as mean ± SEM of three individual mice of one of two independent experiments. p<0.05 for all values compared to non-infected mice.

### Signaling through CCR2, but not CCR5 or Mif, is required for bone marrow to blood egression of CD11b^+^Ly6C^+^ monocytic cells during *T. brucei* infection

**Figure 2:**
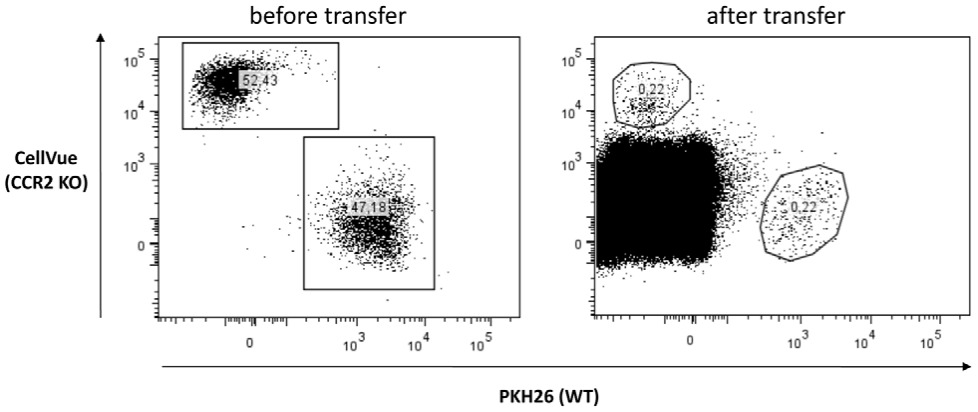
CCR2 does not contribute to liver extravasation of CD11b^**+**^Ly6C^**+**^ monocytic cells during *T. brucei* infection. WT and CCR2 KO CD11b^+^Ly6C^+^ monocytic cells were isolated from bone marrow of mice on day 6 of infection, labeled with PKH26 and CellVue kits, respectively, and injected at a 1-1 ratio in the blood of recipient WT mice on day 6 of *T. brucei* infection. Left panel shows percentages of gated, labeled WT and CCR2 KO cells before injection. 24 hours after transfer liver non-parenchymal were harvested from recipient mice and analysed for the presence of labeled cells. Percentages of gated, labeled WT and CCR2 KO cells are shown (right panel). FACS profiles are representative of one of six animals tested in two independent experiments.

To evaluate whether CCR2, CCR5 or Mif signaling plays a role in the recruitment of CD11b^+^Ly6C^+^ monocytic cells to the liver, CCR2 KO, CCR5 KO, Mif KO mice and their respective WT counterparts were infected with *T. brucei*. In CCR5 KO and Mif KO mice, no difference in the percentage of liver, blood or bone marrow CD11b^+^Ly6C^+^ monocytic cells was observed compared to WT mice on day 6 of *T. brucei* infection (Figure 1B). In infected CCR2 KO mice, the percentage of CD11b^+^Ly6C^+^ monocytic cells was drastically reduced (>75%) as compared to infected WT mice in both the liver and the blood, and coincided with an increase of the percentage of CD11b^+^Ly6C^+^ monocytic cells in the bone marrow (Figure 1B, 1C). Similar modulations were observed for absolute numbers of CD11b^+^Ly6C^+^ monocytic cells in the liver, blood and bone marrow of the respective mouse strains during infection (Figure 1D). These data suggest that the recruitment of CD11b^+^Ly6C^+^ monocytic cells to the liver of *T. brucei* infected mice consists of a two-step process comprising a CCR2-dependent, yet CCR5- and Mif-independent egression step from bone marrow to blood, followed by an extravasation step from blood to liver. Since in CCR2 KO mice CD11b^+^Ly6C^+^ monocytic cells accumulated in the bone marrow, it was unclear whether CCR2 also played a role in blood to liver extravasation during infection. To address this question, CD11b^+^Ly6C^+^ monocytic cells were purified from the bone marrow of *T. brucei* infected WT and CCR2 KO mice, labeled (using PKH26 and CellVue labeling kits respectively), and co-injected at a 1-1 ratio in infected WT recipient mice. Twenty four hours later, liver non-parenchymal cells from recipient mice were analyzed for the presence of labeled cells. As shown in figure 2, the 1-1 ratio of transferred WT to CCR2 KO CD11b^+^Ly6C^+^ monocytic cells could be traced back in infected WT recipient mice, demonstrating that the absence of CCR2 signaling in monocytic cells had no effect on blood to liver migration of CD11b^+^Ly6C^+^ monocytic cells during *T. brucei* infection. To verify whether expression of CCR2 by cells other than CD11b^+^Ly6C^+^ monocytic cells affected the recruitment of the latter cells from the blood to the liver, CD11b^+^Ly6C^+^ monocytic cells isolated from the bone marrow of *T. brucei* infected WT mice and labeled with PKH26 were transferred into infected CCR2 KO or WT recipient mice. The percentage of transferred CD11b^+^Ly6C^+^ cells found back in the liver after 24 hours was not significantly different between CCR2 KO or WT recipients (0.4±0.2% or 0.3±0.1%, respectively). Thus, these data indicate that the CCR2 chemokine receptor signaling in CD11b^+^Ly6C^+^ monocytic cells is crucial for their bone marrow emigration but dispensable for their blood to liver extravasation during *T. brucei* infection.

### Impaired CCR2 signaling reduces liver Tip-DC percentage and pathogenicity during *T. brucei* infection

**Figure 3:**
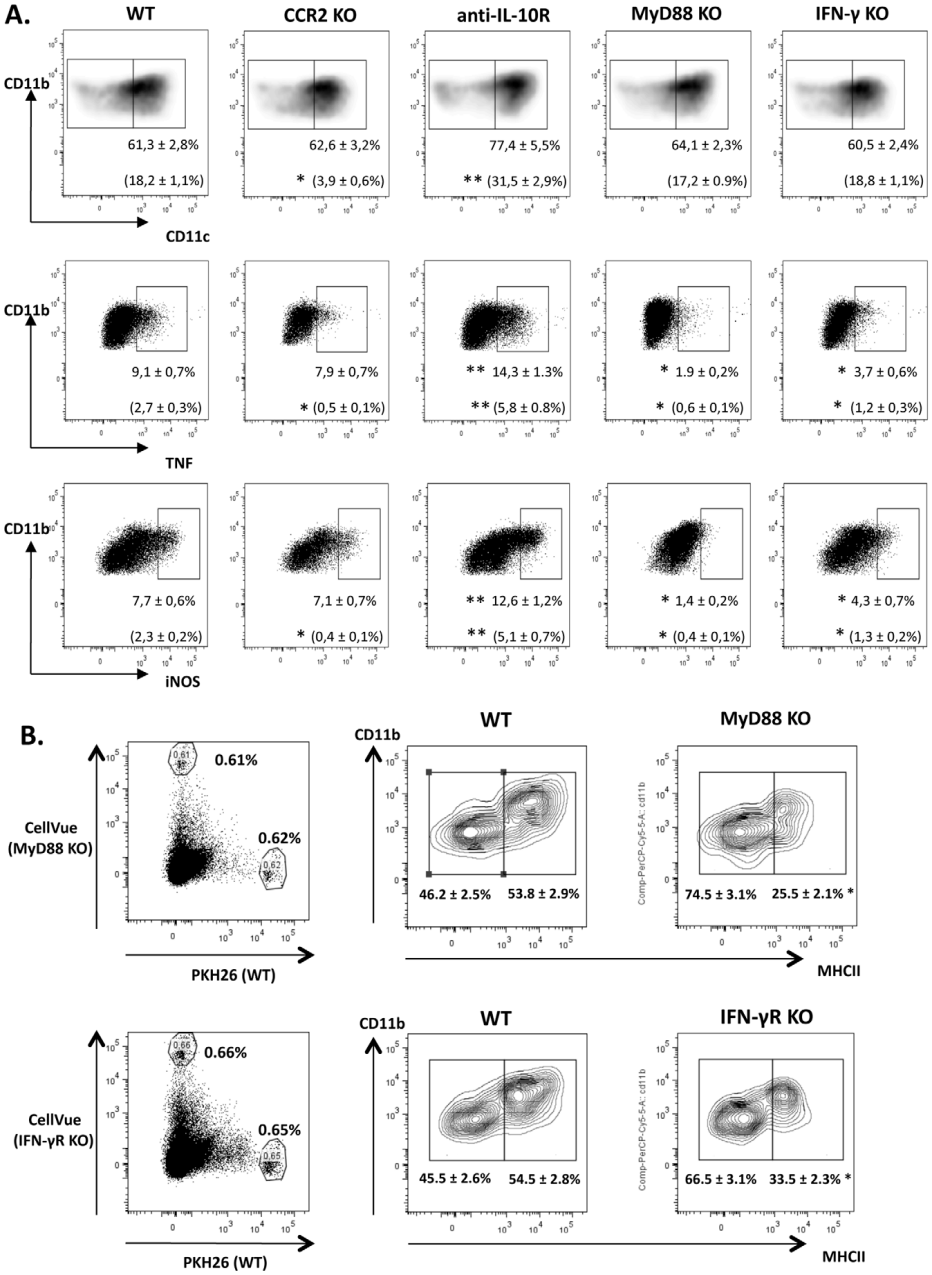
CCR2, IL-10R, MyD88 and IFN-γ signaling differentially contribute to Tip-DC differentiation/maturation during *T. brucei* infection. A) Liver non-parenchymal cells were isolated from WT, CCR2 KO, anti-IL-10R treated WT, MyD88 KO and IFN-γ KO mice on day 6 of infection. CD11b^+^Ly6C^+^ monocytic cells were gated (as indicated in figure 1C) and assayed for co-expression of CD11b and CD11c (top panels), TNF (middle panels) or iNOS (bottom panels). Percentages of CD11c^+^, TNF^+^ or iNOS^+^ cells (rectangular gate) within CD11b^+^Ly6C^+^ monocytic cells are indicated. Corresponding percentages of CD11c^+^, TNF^+^ or iNOS^+^ cells within the total number of living non-parenchymal cells recovered from the liver are indicated between brackets. Data are shown as mean ± SEM of three individual mice of one of three independent experiments performed. FACS profiles are representative of one of nine animals tested in three independent experiments performed. B) CD11b^+^Ly6C^+^ monocytic cells were isolated from bone marrow of WT and MyD88 KO or IFN-γR KO mice on day 6 of *T. brucei* infection, labeled with PKH26 (WT) and CellVue kits (KO), and injected at a 1-1 ratio in the blood of recipient WT mice on day 6 of *T. brucei* infection. Twenty four hours after transfer, liver non-parenchymal were harvested from recipient mice and analyzed for the presence of labeled cells. Percentages of gated, labeled WT and MyD88 or IFN-γR KO cells after transfer are shown (left panels). The expression of MHC-II molecules on the gated labeled cells is shown in the right panels. Data are shown as mean ± SEM of three individual mice of one of three independent experiments performed. FACS profiles are representative of one of four animals tested in three independent experiments. Note that due to the low numbers of transferred cells recovered from the liver, we were unable to assess iNOS or TNF expression on transferred cells using intracellular staining assays. *, significantly lower (p<0.05) and **, significantly higher (p<0.05) compared to WT mice.

**Figure 4:**
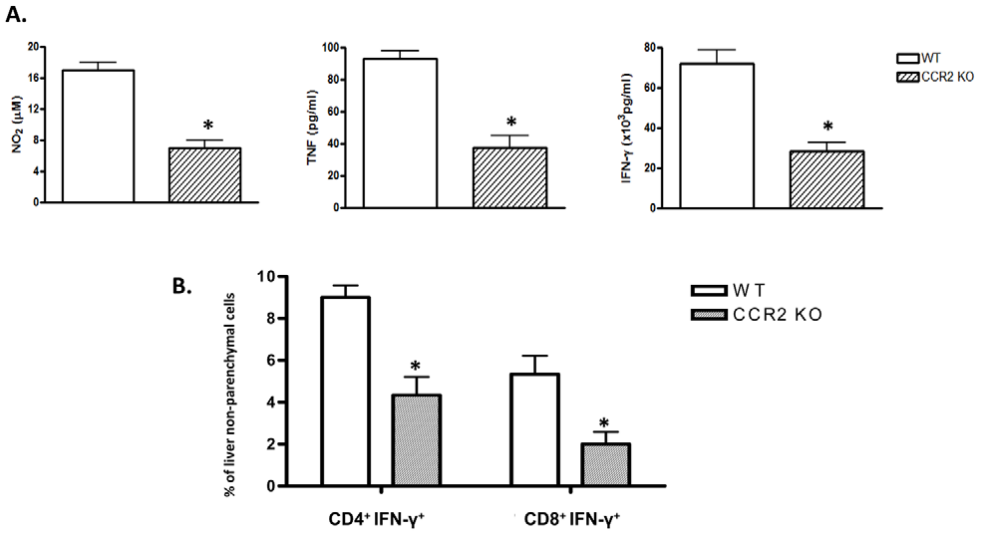
Absence of CCR2 signaling reduces TNF, NO and IFN-γ production during *T. brucei* infection. At day 6 post infection, A) production of TNF, NO_2_ (as measure of NO) and IFN-γ by unstimulated liver non-parenchymal cells after 24 hours of in vitro culture and B) percentages of CD4^+^IFN-γ^+^ and CD8^+^IFN-γ^+^ cells within the liver non-parenchymal cell fraction were determined in WT (white bars) and CCR2 KO (grey bars) mice. Data are shown as mean ± SEM of three individual mice of one of three independent experiments performed. *, significantly (p<0.05) lower compared to WT mice.

During *T. brucei* infection, up to 70% of the CD11b^+^Ly6C^+^ liver monocytic cells can co-express CD11c, classifying them as inflammatory CD11b^+^Ly6C^+^CD11c^+^ dendritic cells [8]. The percentage of CD11c^+^ cells within the CD11b^+^Ly6C^+^ monocytic cell population in the liver of *T. brucei* infected mice was not affected by the absence of CCR2 (Figure 3). Also, the percentage of TNF^+^ and iNOS^+^ cells within CD11b^+^Ly6C^+^ monocytic cells (Figure 3) and their expression levels of co-stimulatory molecules CD80/CD86 and MHC class II (not shown) was similar in WT and CCR2 KO mice, indicating that CCR2 signaling is not involved in the differentiation of CD11b^+^Ly6C^+^ monocytic cells to functional Tip-DCs during *T. brucei* infection. However, in agreement with the reduced percentage of CD11b^+^Ly6C^+^ monocytic cells in the liver of CCR2 KO mice (Figure 1), we observed a significantly reduced percentage of TNF^+^ and iNOS^+^ Tip-DCs within the total liver non-parenchymal cell population of CCR2 KO mice compared to WT mice (Figure 3). The reduced percentage of Tip-DCs in the liver of infected CCR2 KO mice associated with (i) a significant reduction of TNF, NO and IFN-γ secretion in liver non-parenchymal cell cultures and ii) a reduced percentage of liver IFN-γ^+^CD4^+^ and IFN-γ^+^CD8^+^ T cells compared to infected WT mice (Figure 4).

**Figure 5:**
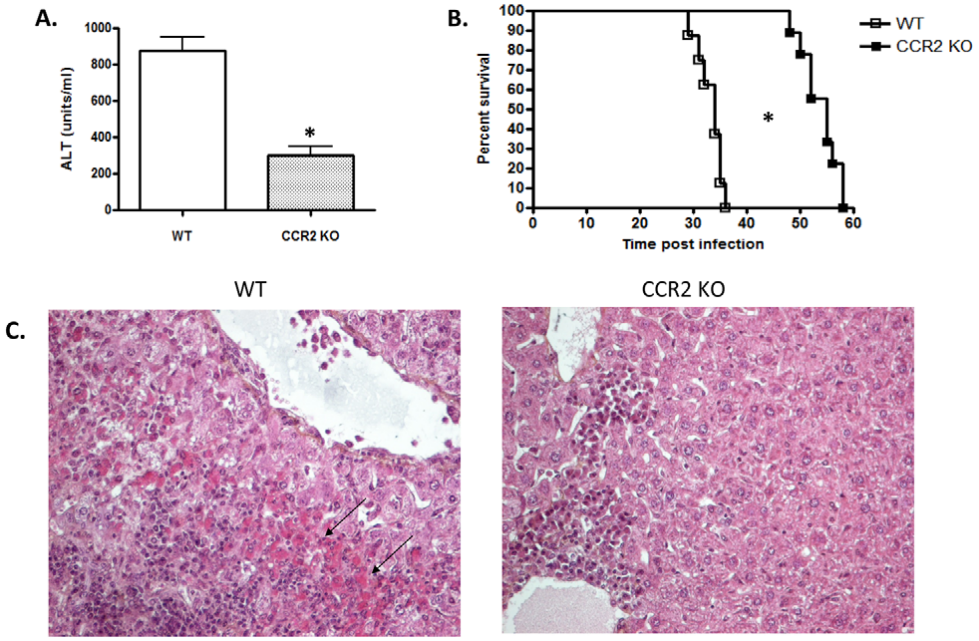
Absence of CCR2 signaling reduces liver pathogenicity and prolongs survival of *T. brucei* infected mice. A) Serum ALT levels were measured in WT and CCR2 KO mice on day 28 of infection (mean ± SEM of three individual mice of one of two independent experiments performed). *, significantly (p<0.05) lower compared to WT mice. B) Survival time of infected WT and CCR2 KO mice. Data are representative of one of two independent experiments performed. *, significantly longer (p<0.05) compared to WT mice. C) Microscopic examination (H&E staining; magnification ×100) of liver sections from WT and CCR2 KO mice on day 28 of infection (representative of 3 animals tested). In CCR2 KO mice hepatocyte necrosis is low, whereas in WT mice important hepatitis and fields of necrosis are observed (arrows).

Although TNF and IFN-γ are involved in the control of parasitemia during *T. brucei* infection [4], [13], parasitemia in WT and CCR2 KO mice was similar indicating that reduced production of TNF and IFN-γ in CCR2 KO mice was still sufficient for efficient parasite control (not shown). On the other hand, the expansion of Tip-DCs and their concomitant production of TNF and NO may contribute to liver injury and reduced survival of *T. brucei* infected mice [8]. In this context, infected CCR2 KO mice exhibited lower levels of serum ALT than infected WT mice on day 28 post infection (Figure 5A), i.e. when the lethal pathogenic features of the disease become apparent. Reduced liver injury in *T. brucei* infected CCR2 KO mice was further confirmed by histological analysis (Figure 5B). Finally, decreased liver injury in the absence of CCR2 signaling correlated with a significantly increased survival time (from 32±3 days in WT mice to 53±4 days in CCR2 KO mice) (Figure 5C). Thus, in the absence of CCR2 signaling, the reduction in the percentage of CD11b^+^Ly6C^+^ monocytic cells and concomitant reduction in Tip-DC percentage in *T. brucei* infected mice lowers the production of TNF and NO in the liver, reduces liver injury while preserving parasite clearing capacity and increases the survival time of the host.

**Figure 6:**
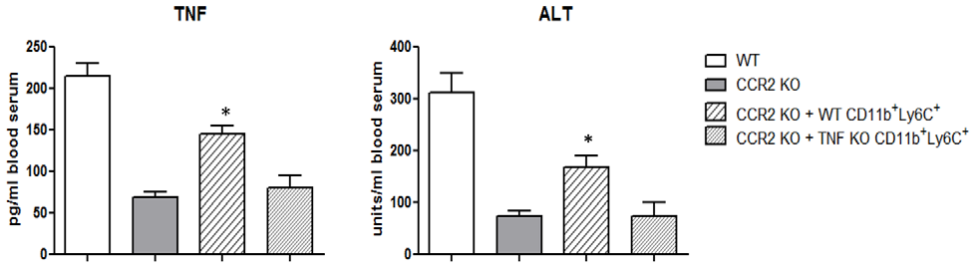
Transfer of CD11b^**+**^Ly6C^**+**^ TIP-DC precursors increases pathogenicity during *T. brucei* infection. CD11b^+^Ly6C^+^ monocytic cells were isolated from the bone marrow of WT or TNF KO mice on day 6 of *T. brucei* infection, labeled with CellVue and transferred intravenously into infected recipient CCR2 KO mice. Twenty four hours after transfer blood serum was analyzed for TNF concentration and ALT activity. Data are mean ± SEM of three individual mice of one of three independent experiments performed. Note that due to the low numbers of transferred cells recovered from the liver, we were unable to assess iNOS or TNF expression on transferred cells using intracellular staining assays. *, significantly higher (p<0.05) compared to CCR2 KO mice that did not receive CD11b^+^Ly6C^+^ monocytic cells.

To further support a role for Tip-DCs in pathogenicity during *T. brucei* infection, we transferred their CD11b^+^Ly6C^+^ monocytic cell precursors purified from the bone marrow of WT infected mice into infected recipient CCR2 KO mice on day 6 post infection. Twenty four hours after transfer a significantly increased concentration of TNF and increased ALT activity in blood serum of recipient CCR2 KO mice was observed (Figure 6). In contrast, transfer of CD11b^+^Ly6C^+^ monocytic cell purified from TNF KO mice affected neither TNF nor ALT levels of recipient CCR2 KO mice (Figure 6). Although up to 52±4% of WT CD11b^+^Ly6C^+^ monocytic cells co-expressed CD11c and MHC-II after transfer, reflecting their differentiation and maturation towards Tip-DCs, TNF and ALT levels in recipient CCR2 KO mice did not reach levels achieved in infected WT mice at the same time point post infection (Figure 6). This may result from the low percentage of transferred cells recovered in the liver (0.9±0.1% of the non-parenchymal cell fraction corresponding to about 1.2±0.2×10^5^ cells), which is likely due to the systemic nature of *T. brucei* infection [14]. These data strongly suggest that Tip-DCs contribute to pathogenicity during *T. brucei* infection.

### IL-10 limits CCL2/CCR2 mediated recruitment of CD11b^+^Ly6C^+^ monocytic cells during *T. brucei* infection

**Figure 7:**
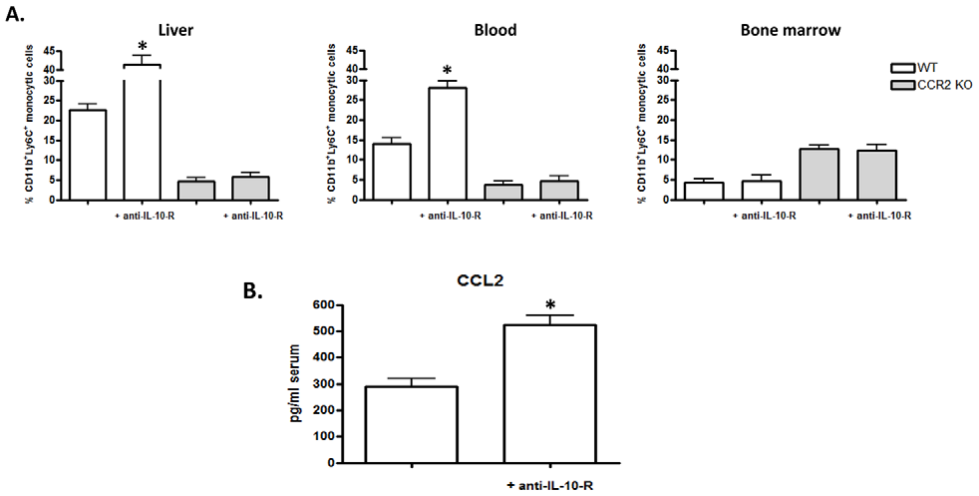
Anti-IL-10R treatment increases CD11b^**+**^Ly6C^**+**^ monocytic cell percentages and serum CCL2 concentration during *T. brucei* infection. WT mice were treated with neutralizing anti-IL-10R antibody or control antibody on days 7 and 9 of infection and euthanized on day 10. A) Percentages of CD11b^+^Ly6C^+^ monocytic cells in the non-parenchymal liver cell fraction, blood and bone marrow of WT (white bars) and CCR2 KO (grey bars) mice with or without anti-IL-10R treatment. B) CCL2 protein concentration in blood sera of WT *T. brucei* infected mice. Data are the mean ± SEM of three individual mice tested in one of three independent experiments performed. *, significantly (p<0.05) higher compared to control treated WT mice.

IL-10 has been shown to limit the generation of Tip-DCs from CD11b^+^Ly6C^+^ cells during *T. brucei* infection [8]. In agreement, treatment with neutralizing anti-IL-10R antibody on days 7 and 9 post infection, i.e. at the peak of IL-10 production [8] increased the differentiation of CD11b^+^Ly6C^+^ monocytic cells towards inflammatory DCs and their subsequent maturation to Tip-DCs in the liver (Figure 3). At this point, it was not established whether IL-10 also directly influenced the recruitment of liver associated CD11b^+^Ly6C^+^ monocytic cells. To consider this possibility, WT and CCR2 KO *T. brucei* infected mice were treated with neutralizing anti-IL-10R antibody. In WT but not in CCR2 KO mice this treatment mimicked the phenotype observed in IL-10 KO mice [8], killing the mice at 11±1 days post infection with increased liver injury (serum ALT levels: 810±68 versus 223±39 U/ml in anti-IL-10R and control antibody treated WT mice at day 10 post infection). In the liver as well as in the blood of infected anti-IL-10R antibody treated WT mice the percentage of CD11b^+^Ly6C^+^ monocytic cells within total liver non-parenchymal cells doubled compared to infected control antibody treated WT mice (Figure 7). In contrast, the percentage of liver and blood CD11b^+^Ly6C^+^ monocytic cells did not change in CCR2 KO mice upon anti-IL-10R antibody treatment, showing that IL-10 regulates peripheral CD11b^+^Ly6C^+^ monocyte percentages in a CCR2-dependent way. Of note, the percentage of bone marrow CD11b^+^Ly6C^+^ monocytic cells did not change in anti-IL-10R treated WT or CCR2 KO mice, suggesting that IL-10 signaling did not alter bone marrow monopoiesis (Figure 7). Interestingly, gene expression level of the main CCR2 ligand Ccl2 (fold induction compared to non infected mice) was further induced in total liver extracts upon anti-IL-10R antibody treatment in infected WT mice (from 5.3±0.9 in control antibody treated mice to 16.4±1.4 in anti-IL-10R antibody treated mice, p<0.05). In addition, blood serum levels of CCL2 increased upon anti-IL-10R treatment (Figure 7). These data indicate that IL-10 limits CD11b^+^Ly6C^+^ monocyte cell recruitment to the liver and the blood during *T. brucei* infection in a CCR2-dependent manner, likely by limiting the production of CCL2.

### IFN-γ and MyD88 signaling induce the generation of Tip-DCs from CD11b^+^Ly6C^+^ monocytic cells during *T. brucei* infection

While the generation of Tip-DCs from liver associated CD11b^+^Ly6C^+^ monocytic cells in *T. brucei* infected mice was impaired by IL-10, the mechanisms triggering this generation are undefined. In this regard, although IFN-γ and MyD88 signaling were documented to contribute to TNF and NO production in *T. brucei* infected mice [5], [15], it is unknown whether IFN-γ and/or MyD88 signaling are involved in Tip-DC differentiation during infection. As shown in Figure 3A, the percentage of CD11c^+^ inflammatory DCs within the liver CD11b^+^Ly6C^+^ population was not affected in the absence of IFN-γ or MyD88 signaling during *T. brucei* infection. However, infected IFN-γ KO and MyD88 KO mice had significantly lower percentages of TNF and iNOS producing cells within the CD11b^+^Ly6C^+^CD11c^+^ inflammatory DCs (Figure 3A). Also, expression levels of co-stimulatory molecules CD80/CD86 and MHC class II were reduced in liver inflammatory DCs from IFN-γ or MyD88 KO compared to WT mice (not shown). To further determine the role of MyD88 and IFN-γ signaling in the generation of Tip-DCs, we co-injected in a 1-1 ratio CD11b^+^Ly6C^+^ monocytes isolated from the bone marrow of *T. brucei* infected WT and MyD88 KO on the one hand, and from infected WT and IFN-γR KO on the other hand, in infected WT recipient mice. Twenty four hours later, the 1-1 ratio of WT versus KO transferred cells was maintained in the liver (Figure 3B), suggesting that IFN-γR or MyD88 signaling was not involved in blood to liver extravasation. In addition, (i) similar percentages of transferred CD11b^+^Ly6C^+^ monocytic cells from WT, IFN-γR KO and MyD88 KO expressed CD11c (54±3%, 52±4% and 50±4% respectively) and (ii) the expression of MHC-II (Figure 3B) and CD80/CD86 (not shown) molecules was significantly reduced on transferred IFN-γR KO and MyD88 KO CD11b^+^Ly6C^+^ monocytic cells compared to WT, confirming our findings in *T. brucei* infected IFN-γ KO and MyD88 KO mice that maturation of CD11c^+^CD11b^+^Ly6C^+^ inflammatory DCs towards a mature Tip-DC phenotype is dependent on both IFN-γ and MyD88 signaling.

Together, these data indicate that IFN-γ and MyD88 signaling are not required for the differentiation of CD11b^+^Ly6C^+^ monocytic cells towards CD11b^+^Ly6C^+^CD11c^+^ inflammatory DCs, but play a role in their further maturation to functional Tip-DCs by inducing the expression of iNOS, TNF and costimulatory molecules.

## Discussion

Murine monocytic cells comprise of distinct functional subsets that can be distinguished based on their expression of the surface markers CD11b, Ly6C, CX3CR1 and CCR2 [16]. One subset consists of CD11b^+^CX3CR1^low^CCR2^high^Ly6C^high^ “inflammatory monocytes”, referred to here as CD11b^+^Ly6C^+^ monocytic cells, which migrate from the bone marrow to inflamed organs following infection [12]. There, inflammatory or pathogen-derived molecules can induce activation of CD11b^+^Ly6C^+^ monocytic cells to CD11c, CD80/86, MHC class II molecule expressing, TNF and iNOS-producing DCs (Tip-DCs) [17]. Tip-DC activity can be beneficial to the host by controlling growth of *Listeria*, *Brucella*, *Leishmania* or influenza virus pathogens [17], [18], [19], [20], however it may also be pro-pathogenic by contributing to tissue damage such as during infection with influenza virus or the African trypanosome *Trypanosoma brucei* [8], [20]. Herein, we have attempted to unravel the pathways underlying the recruitment of CD11b^+^Ly6C^+^ monocytic cells to the liver of *T. brucei* infected mice and the factors regulating their differentiation to Tip-DCs allowing a better understanding of the mechanisms underlying African trypanosomiasis-associated pathogenicity.

Screening of total liver extracts for genes with upregulated expression in *T. brucei* infection yielded chemokine genes previously implicated in monocyte trafficking: *Ccl2* (signaling through CCR2), *Ccl3-4-5* (signaling through CCR5) and *Mif* (signaling through CD74, CXCR2, CXCR4) [21], [22], [23], [24], [25]. FACS analyses revealed that liver CD11b^+^Ly6C^+^ monocytic cells from infected mice expressed high level of CCR2, low level of CCR5 and CD74, and marginal level of CXCR2 and CXCR4 (not shown). No difference in the percentage of CD11b^+^Ly6C^+^ monocytic cells was observed in the bone marrow, blood and liver of infected CCR5 KO or Mif KO mice. In contrast, percentages of CD11b^+^Ly6C^+^ monocytic cells drastically dropped in liver and blood while increasing in bone marrow of infected CCR2 KO mice. Transfer experiments revealed that neither CCR2, nor CCR5 or Mif (not shown) contributed to extravasation of bone marrow-derived CD11b^+^Ly6C^+^ monocytic cells from blood to liver of *T. brucei* infected mice. Together, these data show that CD11b^+^Ly6C^+^ monocyte recruitment to the liver of *T. brucei* parasite infected mice involved an egression step from the bone marrow that is CCR2-dependent, followed by a CCR2-independent extravasation step triggered by yet unidentified factors released by the inflamed tissue, as documented during bacterial infection [22], [26].

CD11b^+^Ly6C^+^ monocytic cells entering the liver of *T. brucei* infected mice first differentiate to CD11b^+^Ly6C^+^CD11c^+^ inflammatory DCs and subsequently maturate to CD80/CD86 high, MHC-II high, TNF and NO secreting Tip-DCs. Both this differentiation and maturation step can be limited by IL-10 [8]. In addition, we show here that treatment of *T. brucei* infected mice with anti-IL-10R antibody strikingly increased the percentage of CD11b^+^Ly6C^+^ monocytic cells in blood and liver but not in the bone marrow, excluding a role for IL-10 on the differentiation of the inflammatory monocyte progenitor (the so-called macrophage and DC precursor, MDP) [27], [28]. However, increased percentage of peripheral CD11b^+^Ly6C^+^ monocytic cells was not observed in CCR2 KO mice treated with anti-IL-10R antibody. In addition, CCL2 protein levels in the blood and gene expression of *Ccl2* in total liver extracts of infected mice was increased by anti-IL-10R treatment during infection. Although many cell types can produce CCL2, we found that *Ccl2* gene expression was induced in liver CD11b^+^Ly6C^+^ monocytic cells during infection and further upregulated upon IL-10 neutralization, suggesting that IL-10 negatively regulates a CCL2 dependent positive feedback loop for liver CD11b^+^LyC6^+^ monocyte recruitment. CD11c^−^ and CD11c^+^ CD11b^+^Ly6C^+^ monocytic cells contributed equally to the induced expression of *Ccl2* (not shown). Finally, the percentage of CD11b^+^Ly6C^+^ monocytic cells was found increased in liver and blood of IL-10 KO mice (not shown), further supporting a role for IL-10 in regulating peripheral CD11b^+^Ly6C^+^ monocytic cell numbers. Taken together, these data suggest that besides impairing differentiation and maturation of CD11b^+^Ly6C^+^ monocytic cells to Tip-DCs, IL-10 can counteract the CCL2/CCR2-mediated recruitment of CD11b^+^Ly6C^+^ monocytic cells/Tip-DCs to liver and blood of *T. brucei* infected mice.

MyD88 and IFN-γ signaling have been implicated in the generation of Tip-DCs in infection models including *Leishmania*, *Brucella* and *Listeria* [18], [19], [29]. Knowing that during *T. brucei* infection, the production of TNF and NO is impaired in MyD88 KO [5] and IFN-γ KO mice (unpublished observation), we investigated whether MyD88 or IFN-γ signaling affected the differentiation/maturation of CD11b^+^Ly6C^+^ monocytic cells to Tip-DCs by using KO mice and co-transfer experiments. The percentage of liver associated CD11b^+^Ly6C^+^ monocytic cells, their extravasation and their differentiation to inflammatory DCs was unaffected by the absence of MyD88 or IFN-γ signaling. On the other hand, inflammatory DCs from infected MyD88 KO mice and IFN-γ KO mice expressed lower levels of CD80/86, MHC class II molecules and produced less TNF and iNOS protein, indicating that MyD88 and IFN-γ signaling are involved in the maturation of inflammatory DCs to functional Tip-DCs. The currently identified *T. brucei*-derived MyD88 signaling triggers, the glycosylphosphatidylinositol-anchored VSG and DNA, inducing the production of TNF and NO, and endotoxin/LPS-like substances from the trypanosome could represent candidates contributing to Tip-DC maturation. While trypanosome DNA has been suggested to interact with TLR9, TLRs that could interact with other trypanosome derived molecules have not been identified. Moreover, it cannot be excluded that increased LPS release into the blood circulation due to secondary bacterial infection and/or increased gut permeability contributes to TLR4-dependent Tip-DC maturation during trypanosome infection [5], [30], [31], [32].

The function of DCs has been poorly examined in the context of African trypanosome infection with the exception of the work from Dagenais et al. showing that splenic conventional CD11c^high^CD8^+^ and CD11c^high^CD8^−^ DCs during *T. brucei (rhodesiense)* infection contribute to activation of VSG-specific Th1 cell responses through the coordinated upregulation of costimulatory molecules, secretion of IL-12, and presentation of VSG peptides to T cells [33]. Conventional DCs in contrast to Tip-DCs are believed not to derive from monocytes [34]. However, Tip-DCs can exhibit T-cell stimulatory capacity [19], [20]. In agreement, in the liver of *T. brucei* infected CCR2 KO mice, reduced percentage of Tip-DCs associated with reduced percentage of IFN-γ producing T cells, referred to previously as “pathogenic” T cells during African trypanosome infection [35]. Our observation that IFN-γ signaling is necessary for the generation of mature functional Tip-DCs in infected mice supports a positive cross-regulation between T cells and Tip-DCs mediated by IFN-γ as was suggested during *Leishmania* infection [19]. In addition to their immunostimulatory function, Tip-DCs may contribute to parasite control during *T. brucei* infection by producing TNF that is essential for this process [4]. Accordingly, *T. brucei* infected MyD88 KO mice and IFN-γ KO mice exhibited reduced Tip-DC percentage and reduced production of TNF, correlating with an inability to efficiently control parasitemia [5]. However, although Tip-DCs and TNF levels were reduced in CCR2 KO mice, control of parasitemia was unaffected. In this respect, it cannot be omitted that the present work only focused on the role of liver associated CD11b^+^Ly6C^+^ monocytic cells while the role of resident liver monocytic cells, including CD11b^−^Ly6C^+^ monocytic cells and CD11b^+^ liver Kupffer cells, remains to be addressed. While absence of CCR2 could only affect recruited CD11b^+^Ly6C^+^ monocytic cells, absence of IFN-γ and MyD88 signaling can impair the activation of both recruited and resident liver monocytic cells during infection. Moreover, the relative contribution of recruited versus resident liver monocytic cells to parasite control and induction of pathogenicity may differ. Indeed, >75% of *T. brucei* are removed from circulation by resident liver Kupffer cells arguing for a major role of these cells in parasite control [36]. On the other hand, a more pro-pathogenic function for recruited CD11b^+^Ly6C^+^ monocytic cells/Tip-DCs is supported by the observation that in CCR2 KO mice (this study) or in *T. brucei* infected mice treated with IL-10 [8], reduced percentage of recruited CD11b^+^Ly6C^+^ monocytic cells/Tip-DCs associated with reduced pathogenicity and increased survival without affecting parasitemia. In the same vein, drastic shortened survival time of *T. brucei* infected IL-10 KO mice due to excessive tissue injury coincided with increased levels of CD11b^+^Ly6C^+^ monocytic cells/Tip-DC in the liver but normal parasite clearance capacity [8], [37]. Finally, our observation that the transfer of CD11b^+^Ly6C^+^ monocytic cells from infected WT mice that can maturate to Tip-DCs [8], but not from TNF KO mice, increased TNF concentration and ALT activity in serum of infected CCR2 KO recipients supports a role for these cells in the induction of liver injury during *T. brucei* infection.

**Figure 8:**
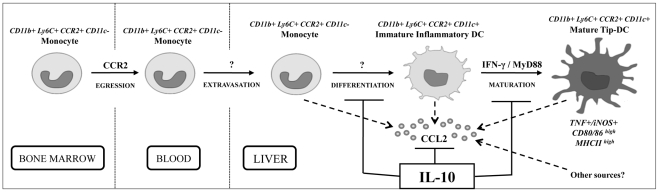
Model for migration, differentiation and maturation of CD11b^**+**^Ly6C^**+**^ monocytes to Tip-DCs during *T. brucei* infection.

In conclusion, we have unraveled the pathways involved in the recruitment of a major pathogenic Tip-DC population in the liver of *T. brucei* infected mice (Figure 8). The development of Tip-DCs is a multi-step process including (i) a CCR2-dependent egression of CD11b^+^Ly6C^+^ inflammatory monocytic cells from bone marrow, followed by (ii) a differentiation step to immature inflammatory DCs (CD11c^+^ but CD80/CD86/MHC-II^low^) which is IFN-γ and MyD88 signaling independent and (iii) a maturation step of inflammatory DCs to functional (CD80/CD86/MHC-II^high^) TNF and NO producing Tip-DCs which is IFN-γ and MyD88 signaling dependent. IL-10 could inhibit the CCL2/CCR2-mediated egression of CD11b^+^Ly6C^+^ monocytic cells from the bone marrow as well as their differentiation and maturation to Tip-DCs in the liver. Liver injury in various etiologies can result from uncontrolled activation of monocyte-derived cells recruited to the liver via CCR2 signaling and can be modulated by IL-10 [38], [39], [40], [41], [42]. African trypanosome infections thus represent a useful model to unravel these activation mechanisms and hereby to identify new tools, including monocyte associated, IL-10 inducible genes we have previously identified [10], [11], to treat hepatic inflammation.

## Materials and Methods

### Ethics statement

The experiments, maintenance and care of mice complied with the guidelines of the European Convention for the Protection of Vertebrate Animals used for Experimental and other Scientific Purposes (CETS n° 123). The experiments for this study were approved by the Ethical Committee for Animal Experiments of the Vrije Universiteit Brussel, VUB, Brussel, Belgium.

### Parasites, mice and infections

*Trypanosoma brucei* AnTat 1.1E parasites were a kind gift from N. Van Meirvenne (Institute for Tropical Medicine, Belgium). Female mice kept under filter barrier were used at 8–12 weeks of age. Wild type C57BL/6 mice were from Harlan, The Netherlands. CCR2 KO, CCR5 KO and Mif KO C57BL/6 mice were kindly provided by Dr. D. Engel, University Clinic of Bonn, Germany, Dr. F. Tacke, RWTH-University Hospital Aachen, Germany and Prof. R. Bucala, Yale University School of Medicine, New Haven, USA). IFN-γ KO, TNF KO and MyD88 KO C57BL/6 mice were bred in our animal facility. Parasites stored at −80°C were used to infect cyclophosphamide treated F1 (C57BL/6× BALB/c) mice bred in-house by i.p. inoculation. At day 4 post infection, mice were bled, and parasites were purified by diethyl-aminoethyl-cellulose chromatography. C57BL/6 mice were then infected i.p. with 2000 purified parasites. Parasitemia was monitored by tail blood puncture. When required, mice were injected i.v. with 100 µl of a 1 mg/ml PBS solution of anti-IL-10R antibody (1B1.3a, kindly provided by Dr. M. Moser and C. Coquerelle, ULB, Gosselies, Belgium with the agreement of Schering-Plough Biopharma) or control antibody (rat IgG1, BD Biosciences) on days 7 and 9 of infection and sacrificed on day 10.

### Isolation of liver, blood and bone marrow immune cells

Liver non-parenchymal cells were isolated as follows. Animals were euthanized (CO2) and livers were perfused through the portal vein with 10 ml of 100 U/ml collagenase type III (Worthington Biochemical Corporation) in HBSS. Then, the liver was minced and incubated in 10 ml of a 100 U/ml collagenase III solution in HBSS (20 min, 37°C). The resulting cell suspension was passed through a 70 µm nylon mesh filter and then washed by adding 30 ml of HBSS supplemented with 2 mM EDTA and 10% FCS followed by centrifugation (300 g, 10 min, 4°C). After erythrocyte lysis, pellet was resuspended in 10 ml of Lymphoprep (Lucron Bioproducts) and overlayed with 10 ml of HBSS supplemented with 2 mM EDTA and 10% FCS. After centrifugation (430 g, 30 min, 17°C), the layer of low-density cells at the interface containing non-parenchymal cells was harvested. CD11b^+^Ly6C^+^Ly6G^+^ liver granulocytes were not retained within the non-parenchymal fractions due to their high density characteristic. Bone marrow cells were isolated from hind leg bone of CO_2_ euthanized animals by perfusion with 20 ml of HBSS supplemented with 10% FCS (HBSS/FCS). The resulting cell suspension was passed through a 100 µm nylon mesh filter, centrifuged (300 g, 10 min, 4°C) and resuspended in HBSS/FCS. Blood was isolated on EDTA by heart puncture of euthanized mice, followed by thorough erythrocyte lysis, centrifugation (300 g, 10 min, 4°C) and resuspension of blood immune cells in HBSS/FCS. Immune cells and used buffers were kept on ice during isolation protocols and subsequent analysis.

### Transfer of monocytes

CD11b^+^Ly6C^+^ monocytes were isolated from the bone marrow of infected WT, CCR2 KO, TNF KO, MyD88 KO or IFN-γR KO mice by MACS purification on day 6 post infection. CD11c^+^ and Ly6G^+^ cells were first depleted via negative MACS selection. CD11b^+^Ly6C^+^ monocytes were then isolated via positive CD11b selection on magnetic separation columns according to the manufacturer's protocol (Miltenyi Biotec) resulting in purity ranging from 90–95%. WT and CCR2 KO or MyD88 KO or IFN-γR KO bone marrow cells were then fluorescently labeled using respectively PKH26 (Sigma-Aldrich) and CellVue membrane labeling kits (Polysciences) according to the manufacturer's protocol. Labeled cells were adoptively transferred at a 1-1 ratio (total of 4×10^6^ cells/mouse) through tail vein injection in infected recipient mice on day 6 post infection. Alternatively, CellVue labeled WT or TNF KO monocytes from infected mice were injected in CCR2 KO mice on day 6 post infection (8×10^6^ cells/mouse). Twenty four hours after transfer the recipient mice were sacrificed and presence of labeled cells was analyzed in the liver non-parenchymal cell fraction.

### Quantification of cytokines and NO_2_

Liver cells were resuspended at 2×10^6^/ml in RPMI 1640 (Gibco) supplemented with 10% FCS, 100 U/ml penicillin, 100 µg/ml streptomycin, 0.1 mM non-essential amino acids, 2 mM L-glutamine, and 5×10^−5^ M 2-mercaptoethanol (all from Invitrogen Life Technologies) and cultured in vitro (37°C). Cytokines were quantified in culture supernatants collected after 2 days with specific sandwich ELISAs for IFN-γ (PharMingen) or TNF (R&D Systems), in accordance to the manufacturers' protocols. NO_2_ quantification was assayed by a standard Griess reaction as described [37].

### FACS analysis

For surface markers, cells were stained for 30 min at 4°C using conventional protocols. Cells were pre-incubated with anti-FcγR Ab (clone 2.4G2) before adding (1 µg/10^6^ cells): FITC-conjugated or APC-conjugated MHC-II (clone M5/114.15.2), PerCP-Cy5.5-conjugated or PE-Cy7 conjugated CD11b (M1/70), FITC-conjugated CD80 (16-10A1), FITC-conjugated CD86 (Gl-1), FITC-conjugated Ly6C (AL-21), PE-conjugated or APC-conjugated CD11c (HL3), PE-conjugated CCR5 (C34-3448), unconjugated CCR2 (MC-21, a gift of Dr Matthias Mack, University of Regensburg, Regensburg, Germany), FITC-conjugated CD74 (In-1). Dead cells were excluded by 7-AAD staining. For intracellular TNF staining, cells were cultured 6 hours in the presence of brefeldin-A (BD Bioscience). For intracellular IFN-γ staining, cells were cultured 2 hours with anti-CD3 before adding brefeldin-A (BD Bioscience). Four hours later, cells were fixed, permeabilised (Fix/Perm kit, eBioscience) and analyzed. Antibodies used for intracellular staining were APC-conjugated TNF (clone MP6-XT22), unlabeled rabbit iNOS (M19) and APC-conjugated anti-rabbit IgG. Cells were analyzed on a *FACSCanto II* and analysis was performed using FlowJo. Antibodies were purchased from BD Biosciences, eBioscience or R&D Systems.

### Gene expression analysis

CD11b^+^ Ly6C^+^ cells were isolated from liver non-parenchymal cells by negative selection for CD4, CD8 and CD19 followed by positive Ly6C selection, using a two-step labeling with Ly6C-PE (BD Bioscience) and anti-PE beads (Miltenyi Biotec) on magnetic separation columns according to the manufacturer's protocol (Miltenyi Biotec) with a purity ranging from 90–95%. Three ×10^6^ purified CD11b^+^Ly6C^+^ liver cells were put in Trizol (Invitrogen) and stored at −80°C. For total liver extracts, pieces of liver (0.5×0.5 cm) were minced, washed with HBSS and after centrifugation put in Trizol. Gene expression in liver extracts or isolated populations was analyzed by quantitative real time PCR using the conditions described [43]. Results of the PCR analyses were normalized against the house-keeping gene S12. Primers used were: *Cxcl-9* (Forward: TCCTTTTGGGCATCATCTTC, Reverse: TTCCCCCTCTTTTGCTTTTT), *Cxcl-10* (Forward: GGATGGCTGTCCTAGCTCTG, Reverse: ATAACCCCTTGGGAAGATGG), *Ccl3* (Forward: CGGAAGATTCCACGCCAATTC, Reverse: GGTGAGGAACGTGTCCTGAAG), *Ccl4* (Forward: GCCCTCTCTCTCCTCTTGCT, Reverse: GTCTGCCTCTTTTGGTCAGG), *Ccl5* (Forward: ACAGGTCAAACTACAACTCCA, Reverse: TCAGCTCTTAGCAGACATTGG), *Ccl2* (Forward: CACTCACCTGCTGCTACTCATTCAC, Reverse: GGATTCACAGAGAGGGAAAAATGG), *Mif* (Forward: CTTTTAGCGGCACGAACGAT, Reverse: AAGAACAGCGGTGCAGGTAA).

### Microscopy, ALT and AST levels

Liver were fixed in Bouin solution (Sigma). Histological sections embedded in paraffin were stained with Hematoxylin-eosin-saffron for microscopic evaluations. Liver alanine aminotransferase (ALT) was measured in serum samples, using commercially available kits (Boehringer Mannheim, Mannheim, Germany).

### Statistical analysis

All comparisons were tested for statistical significance using the unpaired t test with Welch's correction from GraphPad Prism 4.0 software.
